# Periodic nano/micro-hole array silicon solar cell

**DOI:** 10.1186/1556-276X-9-654

**Published:** 2014-12-03

**Authors:** Guan-Yu Lai, Dinesh P Kumar, Zingway Pei

**Affiliations:** 1 Department of Electrical Engineering, National Chung Hsing University, 250 Ku-Kang Rd, Taichung 402, Taiwan; 2 Graduate Institute of Optoelectronic Engineering, National Chung Hsing University, 250 Ku-Kang Rd, Taichung 402, Taiwan; 3 Nanoscience and Nanotechnology Research Center, National Chung Hsing University, 250 Ku-Kang Rd, Taichung 402, Taiwan

**Keywords:** Metal catalyst, Nano/microhole Si array, Reflectance, Efficiency

## Abstract

In this study, we applied a metal catalyst etching method to fabricate a nano/microhole array on a Si substrate for application in solar cells. In addition, the surface of an undesigned area was etched because of the attachment of metal nanoparticles that is dissociated in a solution. The nano/microhole array exhibited low specular reflectance (<1%) without antireflection coating because of its rough surface. The solar spectrum related total reflection was approximately 9%. A fabricated solar cell with a 40-μm hole spacing exhibited an efficiency of 9.02%. Comparing to the solar cell made by polished Si, the external quantum efficiency for solar cell with 30 s etching time was increased by 16.7%.

## Background

Previous studies have reported that nanostructure surfaces can efficiently couple incident light into semiconductors [1-3]. Efficient light harvesting is vital for solar cells [4,5]. In addition to light coupling, efficient carrier transport in a nanowire structure has been suggested to increase the short-circuit current [6-8]. Therefore, several studies on solar cells have employed nanostructures to enhance performance [9-15]. The difficulty in establishing contact in nanostructures limits progress. In one study, a conducting polymer was adopted because it easily filled the space between nanostructures, enabling high efficiency to be achieved [15]. Creating microholes might be an appropriate approach [16]; however, using microholes may cause the advantages of light harvesting to be lost. In this study, we propose a microhole array structure to facilitate fabrication by implementing metal catalyst etching. Inside the hole, we spontaneously produced a nanowire array to achieve low reflectance. The optical reflectance of nano/microhole arrays with various spacings was evaluated. A solar cell was manufactured using this structure to demonstrate the possibility of attaining high efficiency.

## Methods

**Figure 1:**
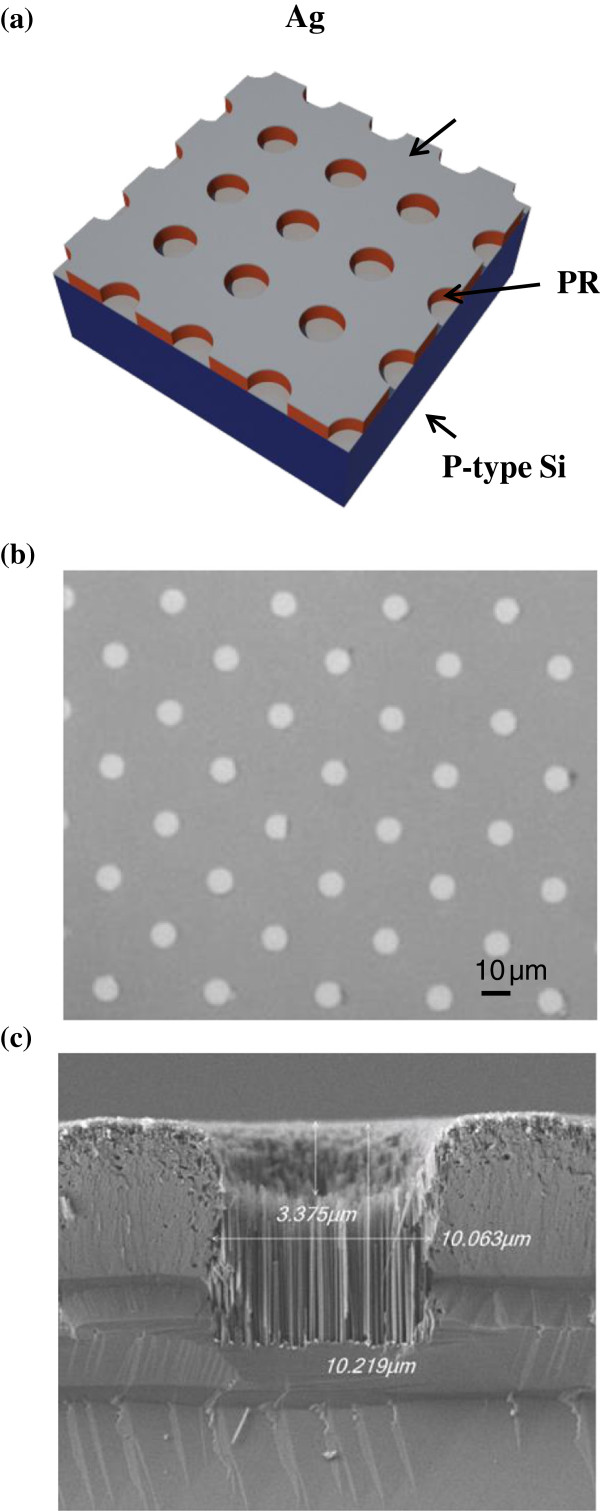
**Schematic structure, optical microscopy, and SEM cross-section photograph. (a)** The schematic structure of lift-off process to make micro/nano-holes by metal catalyst. **(b)** The optical microscopy of the Ag array (10 μm in hole diameter) after lift-off process. **(c)** The SEM cross-section photograph of microhole array. The nanowires were found inside microhole.

The microhole arrays were implemented using metal-catalyst etching on a (100) p-type Si wafer (1 to 10 Ω cm). The wafer was cleaned using acetone in an ultrasonic bath for 10 min. After the substrate was rinsed with deionized (DI) water, it was immersed in a H_2_SO_4_ and H_2_O_2_ mixed solution for 10 min at 120°C. The substrate was further rinsed in DI water, blow-dried, and heated to 120°C in an oven. The microhole array pattern was implemented using photolithography. The diameter of holes on the photomask was 10 μm. The spacing between the holes was varied from 10 to 40 μm. To fabricate the microhole array, a Ag pattern was created by using the lift-off process for catalyst etching. The procedure is described as follows: a layer of photoresist (PR) was spin-coated onto the Si substrate. The Si substrate was then exposed to ultraviolet light through a photomask. After development, the PR on top of the Si substrate was patterned using a periodic hole array. A 50-nm-thick Ag was deposited onto the PR-coated Si substrate. A schematic diagram is shown in Figure 1a. After the PR was removed using acetone, a periodic Ag microdisk array with a diameter of 10 μm was obtained, as shown in Figure 1b. Metal-catalyst etching was conducted by immersing this sample into a H_2_O_2_/HF-mixed solution. Ag has a higher electron affinity than that of Si; therefore, the electrons in Si tend to accumulate on the surface of Ag. The H_2_O_2_ harvests these electrons and becomes H_2_O, as expressed in chemical reaction (1). The Si, lacking electrons, simultaneously reacts with H_2_O to form SiO_2_ and generate electrons, as expressed in chemical reaction (2). The HF then etches the SiO_2_ from the Ag, as expressed in chemical reaction (3). This process consumes no Ag and produces vertical Si patterns.

(1) $$H_{2}O_{2}\;+\;2H^{+}\;+\;2e^{‒}\;\to \;2H_{2}O$$

(2) $$Si\;+\;2H_{2}O\;\to \;{SiO}_{2}\;+\;4H^{+}\;+\;4e^{‒}$$

(3) $${SiO}_{2}\;+\;6HF\;\to \;H_{2}{SiF}_{6}\;+\;2H_{2}O$$

A 10-μm-deep microhole after being etched for 15 min is illustrated in the cross-sectional scanning electron microscopy (SEM) image shown in Figure 1c. As shown in Figure 1c, the diameter of the hole is 10 μm. Inside the hole, Si nanowires were clearly observed. After being etched, the Ag was removed using a HCl/HNO_3_ (3:1 (*v*/*v*)) mixed solution. The structure was named ‘nano/microhole array.’ The n + emitter in a solar cell was fabricated by spraying H_3_PO_4_ onto a p-Si wafer and then annealing the sample in a furnace at 900°C for 30 min [17]. The doping concentration of phosphorus was calculated by converting the resistivity of Si into carrier concentration, in which the resistivity of Si was measured by a spreading resistance profiler. By doping concentration profile, the phosphorus diffuse into Si around 0.2 μm. The solar cells were then implemented by depositing top and bottom electrodes. The area of the solar cell was 1.0 cm^2^.

## Results and discussion

**Figure 2:**
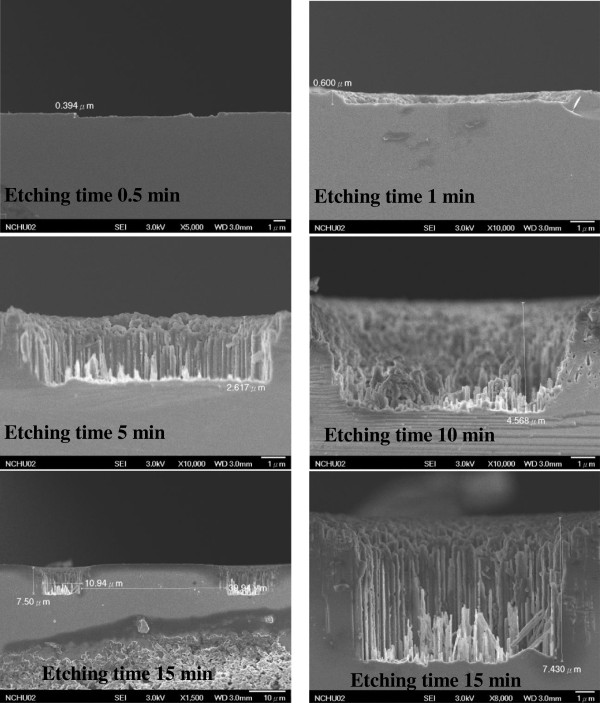
**The cross-sectional SEM images for the nano/micro-hole array.** Etching time of 0.5, 1, 5, 10, and 15 min.

**Figure 3:**
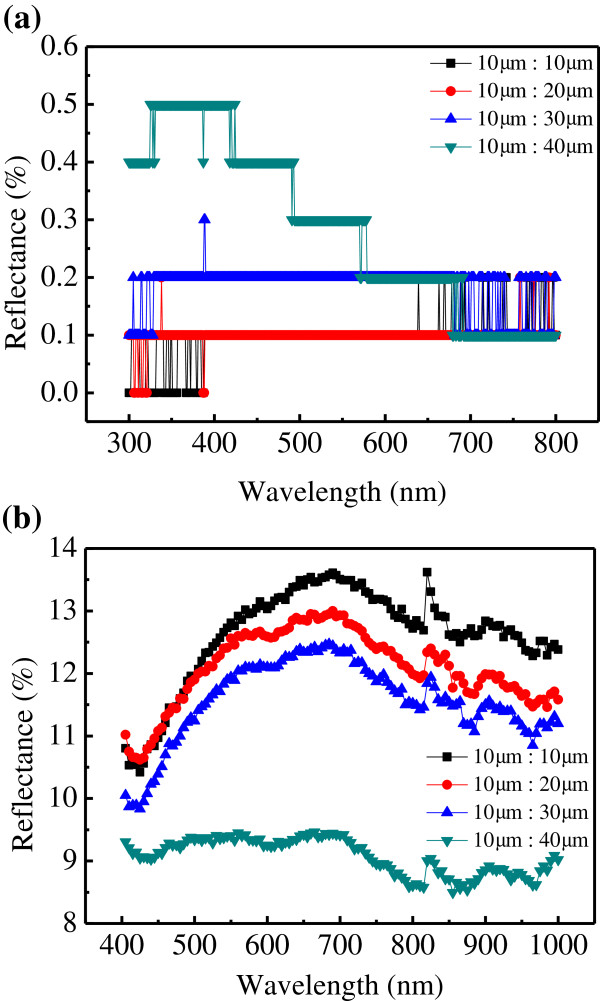
**Specular and total reflectance of the nano/microhole array. (a)** The specular reflectance of the nano/microhole array for different hole spacing. **(b)** The total reflectance of the nano/microhole array for different hole spacing. The etching time is 15 min.

Cross-sectional SEM images of a nano/microhole array at various etching times are shown in Figure 2. The depths of the hole after 0.5, 1, 5, 10, and 15 min were 0.394, 0.6, 2.6, 4.57, and 7.5 μm, respectively. The etching rate was approximately 0.5 μm/min. The specular optical reflectance on nano/microhole arrays with various spacings was measured at a wavelength of 300 to 800 nm. The specular reflectance was well below 1% for all of the samples, as shown in Figure 3a. The occurrence of the step-like reflectance is due to the resolution limit of the measurement, which is 0.1%. The microhole array sample with highest hole density (10-μm-spacing) exhibits lowest reflectance. In addition to the specular reflectance, the textured surface may cause the incident light scattered back to the incident plane, called diffuse reflectance. The total reflectance (*R*_tot_) including specular and diffuse reflectance was measured using an integrated sphere, as shown in Figure 3b. The highest *R*_tot_ was approximately 14% for the 10-μm-spacing sample, whereas that of the 40-μm-spacing sample was 9%. The solar spectrum-related reflectance (*R*_sol_) was 12.66%, 12.16%, 11.65%, and 9.21% for the samples with spacings of 10, 20, 30, and 40 μm, respectively. The *R*_sol_ was determined using the Equation (4):

(4) $$R_{\mathrm{sol}}=\frac{\int_{300}^{800}R_{\mathrm{tot}}\left(\lambda \right)\cdot I\left(\lambda \right)\cdot d\lambda }{\int_{300}^{800}I\left(\lambda \right)\cdot d\lambda }$$

where *I*(*λ*) is the wavelength-dependent solar irradiance.

**Figure 4:**
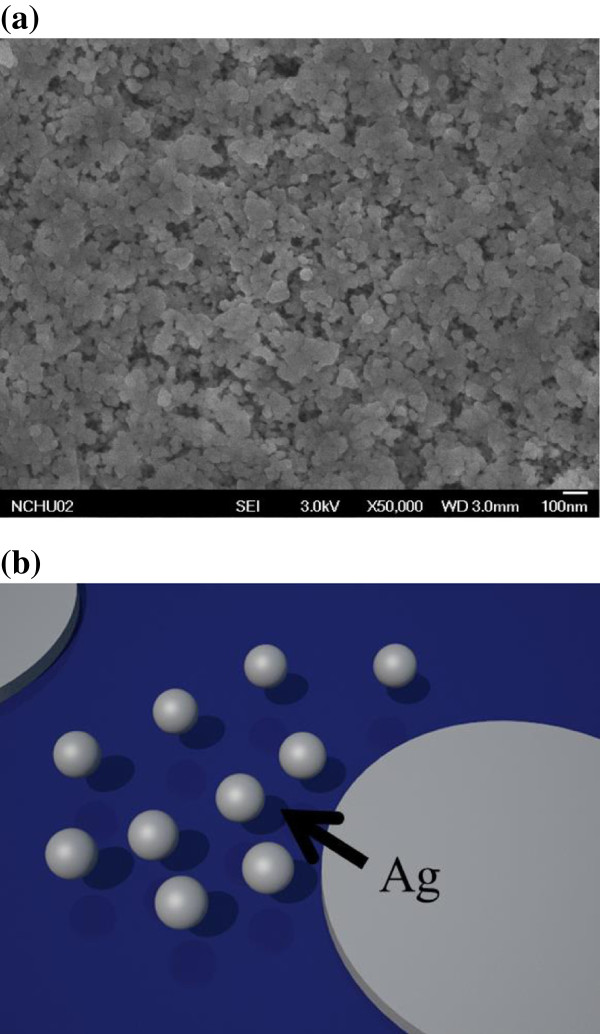
The SEM of the Si surface (a), and (b) the schematic presentation of Ag dissociation in the solution.

**Figure 5:**
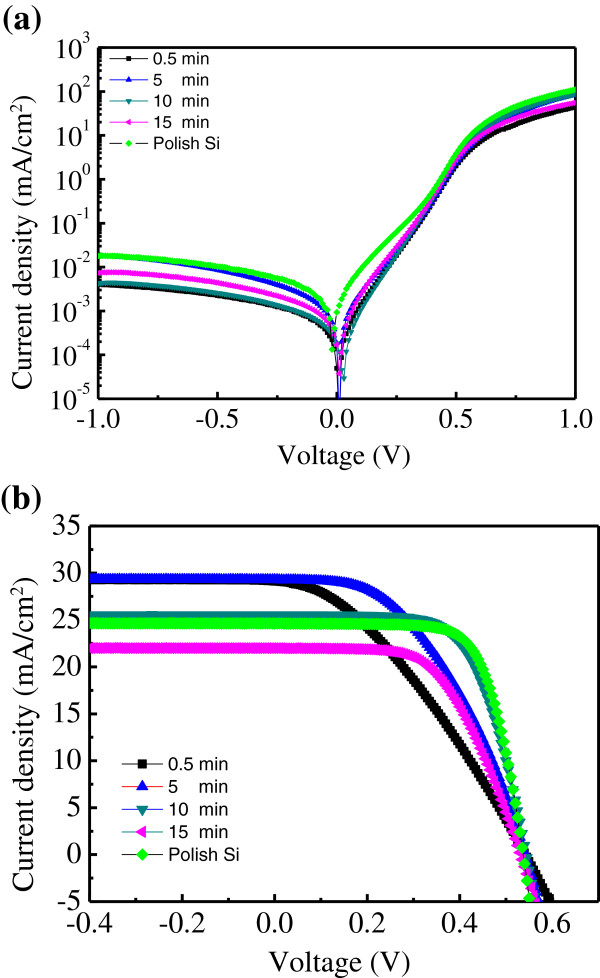
**Dark current–voltage and photovoltaic characteristics for the flat Si and etched Si solar cell. (a)** The dark current–voltage characteristics for the flat Si and etched Si solar cell. **(b)** The photovoltaic characteristics for the flat Si and etched Si solar cell. The etching time ranged from 0.5 to 15 min.

**Figure 6:**
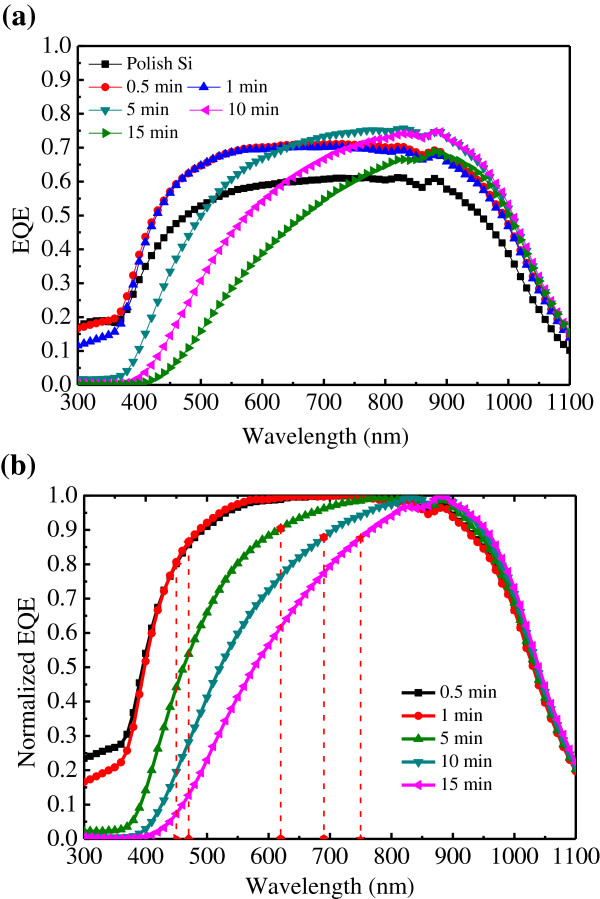
**External and normalized quantum efficiency of the solar cell. (a)** The external quantum efficiency of the solar cell with different etching time. **(b)** The normalized external quantum efficiency of the solar cell with different etching time. The etching time ranged from 0.5 to 15 min.

Unexpectedly, the nano/microhole array with a large surface area (40-μm spacing) exhibited a low reflectance. This is not usual because a flat surface will cause specular reflectance only. The higher diffuse reflectance indicates the surface without hole array may not be flat. To prove this assumption, surface picture was taken by plan-view SEM. Figure 4a shows a SEM image of the Si surface. A porous surface was observed on the area that was not covered by Ag. The incoming light was scattered, causing the specular reflectance to be well below 1%. In addition, the porous-like surface scattered less incoming light than did the periodic structure. Therefore, the sample with a spacing of 40 μm exhibited the least reflectance and is suitable for solar cell application. The mechanism that caused the development of the porous-like surface might be the dissociation of Ag in the H_2_O_2_/HF solution. During the etching process, Ag atoms can be converted into Ag^+^ ion by H_2_O_2_. The Ag^+^ ion can be recovered back to Ag by taking electrons from silicon. This ionized and recovered process will lead Ag to diffuse up and renucleate on the undesigned silicon surface to form new etching sites [18]. A schematic diagram of Ag dissociation in the solution during etching is illustrated in Figure 4b. The dissociation of Ag produced a nanowire in the microholes as well as in the undesigned area. The photovoltaic characteristics were measured under a AM 1.5G condition by using a solar simulator (Model XES-40S1, San-EI Electric Co., Ltd., Osaka, Japan) equipped with an Agilent B2912A I-V meter (Agilent Technologies, Santa Clara, CA, USA). The dark current–voltage of the nano/microhole array solar cell with a hole spacing of 40 μm at various etching times is shown in Figure 5a. No extensive dark current–voltage difference between the samples was observed. The photocurrent-voltage characteristics of the nano/microhole array solar cell are shown in Figure 5b. The device labeled ‘polish-Si’ was a reference device for which no nano/microhole array was composed. This device exhibited a 24.6-mA/cm^2^ short-circuit current density (*Jsc*), 537-mV open circuit voltage (*Voc*), and 0.7 fill factor (*FF*). The power conversion efficiency (*η*) was therefore 9.37%. By using metal-catalyst etching, the short-circuit current increased extensively. After only 0.5 min of etching, the *Jsc* increased to 29.1 mA/cm^2^; this increase can be attributed to the reflectance reduction after etching. However, the porous-like surface rendered achieving favorable electrical contact difficult. The high contact resistance caused the *FF* to be low. Consequently, a low efficiency of 5.59% was obtained. After the etching time was increased to 5 min, the *Jsc* did not increase. The contact resistance was reduced, possibly because the nano/microhole array provided a large surface area for contact. The *Jsc* decreased to 25.5 mA/cm^2^ as the etching time was increased to 10 min. This result is discussed in the subsequent paragraph. In a deep hole, the contact resistance substantially decreased. The series resistance reduced to 5.6 Ω, which is near the value of polish-Si. In addition, the *FF* improved to 0.65 and the efficiency increased to 9.02%. After the etching time was increased further to 15 min, the efficiency decreased to 6.9%. Both *Jsc* and *FF* decreased. The decreased FF might be explained by the suddenly increased *Rs*. The *Rs* is expected to reduce as the hole-depth increase by the larger contact area. However, the formation of metal contact in this experiment is not by the metal paste, it is approximately 100 nm thick deposited by thermal evaporator. Therefore, in the deep hole, the metal line might be broken that causes the increased *Rs*. Table 1 lists details on the photovoltaic parameters. To explore the unusual trend in the *Jsc*, external quantum efficiency (EQE) was measured. The EQE spectrum for the nano/microhole array solar cells at various etching times is shown in Figure 6a. The EQE for polish-Si was approximately 0.6 at a wide wavelength range and increased to 0.7 at an etching time of 0.5 min. However, the spectrum indicated that the EQE at a short wavelength decreased as the etching time was further increased, possibly because of carrier recombination at porous-like layers. The depth of the porous-like layer increased as the etching time was increased. The absorption of the incident light by the Si can be evaluated using Equation (5):

**Table 1:** The photovoltaic parameters for the nano/micro hole array Si solar cell with different etching times

**Etching time (min)**	**Jsc (mA/cm**^**2**^**)**	**Voc (mV)**	***FF***	***η *** **(%)**	**Rs (Ω)**	**Rsh (Ω)**
Polish-Si	24.6	537	0.71	9.37	4.4	8,620
0.5	29.1	542	0.35	5.59	14.9	271
5	29.3	542	0.47	7.40	8.7	2,272
10	25.5	544	0.65	9.02	5.6	6,667
15	22.9	534	0.56	6.90	9.5	4,237

(5) $$I\left(d,\lambda \right)=I\left(0,\lambda \right)\cdot \exp\left(-\alpha \left(\lambda \right)\cdot d\right)$$

where *I*(*d*, *λ*) refers to the intensity of light of wavelength *λ* transmitted into Si at depth *d*; *I*(0, *λ*) refers to the intensity of the incident light at wavelength *λ*; and *α*(*λ*) denotes the absorption coefficient of Si at wavelength *λ* in the unit 1/cm [19]. This equation indicated that a large amount of incident light is absorbed at depth *d* = 1/*α*. This depth was called the absorption depth. The depth of the hole at a specific etching time and the corresponding wavelength at the same absorption depth are listed in Table 2. Because the carriers generated by incident light at the depth of the porous-like layer have a high recombination rate, the EQE at a corresponding wavelength must be reduced. This assumption was explored using a normalized EQE spectrum. Figure 6b shows the normalized EQE for various etching times. The depth corresponding to the wavelength presented in Table 2 fits the reduction point of the EQE satisfactorily. The results indicated that the *Jsc* can be further increased by appropriately passivating the porous-like layer by using methods such as oxidation. If the surface is well passivated, the optimum depth of hole is suggested to be approximately 20 to 25 μm by taking the light absorption edge of Si is approximately 900 to 950 nm.

**Table 2:** The depth of hole at specific etching time and the corresponding wavelength with the same absorption depth

	**Etching Time (min)**				
	**0.5**	**1**	**5**	**10**	**15**
Depth (μm)	0.394	0.6	3.06	5.19	7.5
Wavelength (nm)	450	470	620	690	750

## Conclusions

In this study, we demonstrated the formation of a nano/microhole array in Si by using a simple metal-catalyst etching method. The specular reflectance of this structure can be as low as 1%. The solar spectrum-related total reflection was approximately 9% for the 40-μm spacing sample. Efficiency of 9.02% was achieved by using this nano/microhole array without a surface passivation layer.

## Competing interests

The authors declare that they have no competing interests.

## Authors’ contributions

ZP conceived of the study and participated in its design and coordination. GYL carried out the experiments on fabrication and SEM/optical measurements of the nano/microhole array and solar cell. DK contributed the mechanism discussion and drafted the partially manuscript. ZP revised the manuscript. All authors read and approved the final manuscript.

## References

[B1] Raut HK Ganesh VA Nair AS Ramakrishna S Anti-reflective coatings: a critical, in-depth review Energy Environ Sci 2009 4 3779

[B2] Pei TH Thiyagu S Pei Z Ultra high-density silicon nanowires for extremely low reflection invisible regime Appl Phys Lett 2011 99 153108 10.1063/1.3650266

[B3] Thiyagu S Devi BP Pei Z Chen YH Liu JC Ultra-low reflectance, high absorption microcrystalline silicon nano-stalagmite (μc-SiNS) Nanoscale Res Lett 2012 7 171 10.1186/1556-276X-7-171 22394991 PMC3310714

[B4] Hu L Chen G Analysis of optical absorption in silicon nanowire arrays for photovoltaic applications Nano Lett 2007 7 11 3249 10.1021/nl071018b 17927257

[B5] Muskens OL Rivas JG Algra RE Bakkers M Lagendijk A Design of light scattering in nanowire materials for photovoltaic applications Nano Lett 2008 8 9 2638 10.1021/nl0808076 18700806

[B6] Kayes BM Atwater HA Lewis NS Comparison of the device physics principles of planar and radial p-n junction nanorod solar cells Appl Phys Lett 2005 97 114302

[B7] Pei Z Chang ST Liu CW Chen YC Numerical simulation on the photovoltaic behavior of an amorphous-silicon nanowire-array solar cell IEEE Electron Device Lett 2009 30 1305 1307

[B8] Kumar D Srivastava SK Singha PK Husainb M Kumar V Fabrication of silicon nanowire arrays based solar cell with improved performance Sol Energy Mater Sol Cells 2011 95 215 10.1016/j.solmat.2010.04.024

[B9] Peng K Xu Y Wu Y Yan Y Lee ST Zhu J Aligned single-crystalline Si nanowire arrays for photovoltaic applications Small 2005 1 1062 10.1002/smll.200500137 17193395

[B10] Fang H Li X Song S Xu Y Zhu J Fabrication of slantingly-aligned silicon nanowire arrays for solar cell applications Nanotechnology 2008 19 255703 10.1088/0957-4484/19/25/255703 21828663

[B11] Stelzner T Pietsch M Andrä G Falk F Ose E Christiansen SH Silicon nanowire-based solar cells Nanotechnology 2008 19 295203 10.1088/0957-4484/19/29/295203 21730599

[B12] Thiyagu S Devi BP Pei Z Fabrication of large area high density, ultra-low reflection silicon nanowire arrays for efficient solar cell applications Nano Res 2011 4 11 1136 10.1007/s12274-011-0162-5

[B13] Jung JY Guo Z Jee SW Um HD Park KT Hyun MS Yang JM Lee JH A waferscale Si wire solar cell using radial and bulk p–n junctions Nanotechnology 2010 21 445303 10.1088/0957-4484/21/44/445303 20935359

[B14] Huang BR Yang YK Lin TC Yang WL A simple and low-cost technique for silicon nanowire arrays based solar cells Sol Energy Mater Sol Cells 2012 98 357

[B15] Thiyagu S Hsueh CC Liu CT Syu HJ Lin TC Lin CF Hybrid organic–inorganic heterojunction solar cells with 12% efficiency by utilizing flexible film-silicon with a hierarchical surface Nanoscale 2014 6 3361 10.1039/c3nr06323b 24522339

[B16] Chang YA Li ZU Kuo HC Lu TC Yang SF Lai LW Lai LH Wang SC Efficiency improvement of single-junction InGaP solar cells fabricated by a novel micro-hole array surface texture process Semicond Sci Technol 2009 24 085007 10.1088/0268-1242/24/8/085007

[B17] Bouhafs D Moussi A Boumaour M Abaïdia SEK Mahiou L N^+^ silicon solar cells emitters realized using phosphoric acid as doping source in a spray process Thin Solid Films 2006 510 325 10.1016/j.tsf.2006.01.005

[B18] Zhong X Qu Y Lin YC Liao L Duan XF Unveiling the formation pathway of single crystalline porous silicon ACS Appl Mater Interfaces 2011 3 261 10.1021/am1009056 21244020 PMC3061564

[B19] Green MA Keevers M Optical properties of intrinsic silicon at 300 K Prog Photovolt 1995 3 189 10.1002/pip.4670030303

